# Describing the Myths and Misconceptions Regarding COVID-19 Vaccines Among the Population of the Kingdom of Saudi Arabia

**DOI:** 10.7759/cureus.25932

**Published:** 2022-06-14

**Authors:** Nour B Odeh, Tala H Sriwi, Lana M Arbili, Tarek Z Arabi, Belal N Sabbah, Mohamad S Alkodaymi

**Affiliations:** 1 College of Medicine, Alfaisal University, Riyadh, SAU

**Keywords:** pandemic, misconceptions, vaccines, public health, covid-19

## Abstract

Objectives

This study aims to describe the common myths and misconceptions in addition to the perception and attitudes toward coronavirus disease 2019 (COVID-19) vaccines in the Saudi Arabian community.

Methods

This is a cross-sectional study that included adults (18 years and older) residing in the Kingdom of Saudi Arabia. Participants were asked to complete an online survey that evaluated their perception and attitudes toward the available COVID-19 vaccines in Saudi Arabia. Statistical comparison between two groups and more was done using chi-square, independent t-test, and one-way ANOVA.

Results

A total of 471 responses were analyzed with a majority (83.2%) believing that vaccinations are important. The vaccine preferred among the Saudi Arabian population was Pfizer (65.4%). More than half of our respondents (54.8%) strongly agreed that COVID-19 vaccines can reduce the severity of the COVID-19 infection. Respondents in the healthcare sector were significantly more likely to have a more positive view on vaccines compared to those in non-healthcare sectors (p < 0.001).

Conclusion

The Saudi Arabian population has shown substantial awareness about COVID-19 vaccines; however, public health officials need to further increase awareness measures on COVID-19 vaccines to limit myths and misconceptions, especially among certain populations that are more prone to it.

## Introduction

In December 2019, a novel virus known as the SARS-COV-2 emerged in Wuhan, China. This novel virus caused coronavirus disease 2019 (COVID-19) disease in infected individuals, leading to symptoms such as fever, cough, headache, sore throat, loss of smell and taste, and, in severe cases, shortness of breath. As of November 1, 2021, COVID-19 had a mortality rate of approximately 2% worldwide [1] and 1.6% in the Kingdom of Saudi Arabia (KSA). Severe cases of COVID-19 could lead to hospitalization, critical care admission, or even death. On March 11, 2020, the World Health Organization (WHO) declared COVID-19 a pandemic, and a global initiative has been ongoing to control the spread of the virus through multiple strategies.

Currently, one of the major ongoing strategies in the KSA is to distribute vaccines effective in preventing the disease and its transmission to the public. The vaccines currently available in the KSA are Pfizer-BioNTech (approved on December 10, 2020), Oxford-AstraZeneca (approved on February 18, 2021), and Moderna (approved on July 9, 2021) [2-4]. These vaccines are available throughout the kingdom free of charge, and multiple vaccination centers are operating throughout the region [5]. However, many people are reluctant to take vaccine doses due to the numerous myths and misconceptions surrounding them [6].

In the Arab region, some myths and misconceptions about these vaccines are that they cause infertility, contain a microchip, or even change your DNA [6]. Many of these myths and misconceptions stem from the fact that all available COVID-19 vaccines went through emergency approval and did not have the average amount of testing and time that every other vaccine had to endure [7]. The fastest vaccine ever released was the mumps vaccine, which took about four years, compared to COVID-19 vaccines, which took less than a year [8]. This has caused unrest among the population and can be seen manifested in spreading misconceptions [9]. This study aims to identify the myths and misconceptions that prevent the general Saudi population from getting vaccinated, describe the perception, attitudes, and awareness of the population about the COVID-19 vaccine, as we believe that it is important to aid public health officials in debunking these falsifications, and, therefore, encourage and educate people on how imperative it is to get vaccinated against COVID-19.

## Materials and methods

In this study, an online questionnaire was created through Google Forms (Google, Mountain View, CA) and a URL was distributed to participants for two weeks between 28 July and 13 August 2021, through several social media applications such as Facebook, Twitter, WhatsApp, and Instagram. The study included adults (aged 18 years or older) living in the KSA.

The questionnaire consists of 15 questions related to perception and attitudes toward the available COVID-19 vaccines among the Saudi Arabian population (Pfizer-BioNTech and Oxford AstraZeneca). The Moderna vaccine was not included in the questionnaire, as it was approved after the questionnaire circulation period had ended. Since SARS-CoV-2 is a novel virus and a vaccine against it is newly developed, a lot of myths and misconceptions regarding it have been circulating on social media. Participants were asked to answer the questions based on their general beliefs about vaccination, as well as their perception of the COVID-19 vaccines available in the KSA. They were also asked if they believe common misconceptions and myths that circulate among the population and on social networks. The questionnaire was developed in English and Arabic after reviewing previously published research on this topic along with consulting a panel of experts consisting of two public health specialists and two cross-sectional research experts to confirm content validity [10-14]. To test for clarity and face validity, the questionnaire was piloted in both languages in a group composed of 15 participants who were excluded from participating in the final study. Cronbach's alpha was used to assess the reliability of the internal consistency of the questionnaire.

The questionnaire was only aimed at adults (aged 18 and above) residing in the KSA, and all participants who did not meet those requirements were excluded from the study. Based on published literature [15] and the estimated KSA population of 26,456,921 [16], we calculated our target sample to be 385 to achieve a 95% confidence level with a 5% confidence interval.

The questionnaire was voluntary, confidential, and anonymous, allowing only the principal investigators (PI) and co-PI access to the responses. Participants indicated their age, sex, city of residence, nationality, level of education, and socioeconomic status. The survey had a total of 15 closed-ended multiple-choice questions (Appendix). We asked participants to answer these questions based on their perception of the COVID-19 vaccine and if they believe common misconceptions and myths. The questionnaire also inquired about a general awareness of the COVID-19 vaccines.

Ethical approval was obtained from the Institutional Review Board of Alfaisal University (approval number: IRB-20124). No identifying information was collected to maintain the anonymity of respondents.

Statistical analysis

Data were extracted independently from the online survey and then manually filtered to follow the inclusion criteria. Nominal and categorical data were entered, and statistical analysis was performed using SPSS software version 23 (IBM Corp., Armonk, NY). The frequency of responses was tabulated for each variable. Means and standard deviations were calculated for the five-point Likert scale questions. Furthermore, comparisons between two groups and more were made using the chi-square test, independent t-test, and one-way analysis of variance (ANOVA) with Tukey's honest significant difference (HSD) post hoc test. Statistical significance was determined with a P-value < 0.05, and Cronbach’s alpha coefficient values between >0.7 and <0.9 were deemed to be adequately reliable for comparisons [17].

## Results

Cronbach’s alpha for the questionnaire was 0.835, indicating high internal consistency reliability. We received a total of 500 responses, and after excluding respondents less than 18 years and over 65 years of age, we analyzed 471 responses. The baseline characteristics of survey respondents are depicted in Table 1.

**Table 1:** Baseline characteristics of the survey respondents (n = 471) SAR: Saudi Arabian Riyal.

Sample characteristics	No. (%)
Gender	
Male	214 (45.4%)
Female	257 (54.6%)
Age (years)	
18-24	239 (50.7%)
25-34	51 (10.8%)
35-44	106 (22.5%)
45-54	62 (13.2%)
≥55	13 (2.8%)
Nationality	
Saudi	181 (38.4%)
Non-Saudi	290 (61.6%)
City of residence	
Riyadh	429 (91.1%)
Other	42 (8.9%)
Do you work or study in the healthcare field?	
Yes	252 (53.5%)
No	219 (46.5%)
Highest degree completed	
Have not completed a high school diploma	4 (0.9%)
High school diploma	187 (39.7%)
Bachelor’s degree	190 (40.3%)
Post-graduate degree	90 (19.1%)
Household income	
<3,000 SAR	39 (8.3%)
3,000-9,999 SAR	55 (11.7%)
10,000-25,000 SAR	141 (29.9%)
>25,000 SAR	236 (50.1%)
Are you up to date with all your childhood vaccinations?	
Yes	414 (87.9%)
No	9 (1.9%)
I do not know	48 (10.2%)
Do you usually take the seasonal influenza vaccine (flu shot)?	
Yes	83 (17.6%)
Sometimes	113 (24.0%)
No	275 (58.4%)

Most of our respondents (83.2%, n = 392) believed that vaccinations are important. When asked what their most trusted source of information on the COVID-19 vaccine was, 86.4% (n = 407) responded “The Saudi MOH.” Other trusted sources of information on the COVID-19 vaccine were ranked from most to least picked, including trusted doctors (36.1%, n = 170), awareness campaigns (25.9%, n = 122), social networks (15.1%, n = 71), television (10.8%, n = 51), international health organizations (5.7%, n = 27), and lastly scientific research articles and websites (5.7%, n = 24). Almost all our participants (91.5%, n = 431) reported that they had already received at least one dose of the vaccine; meanwhile, only 1.3% (n = 6) do not intend to take the vaccine. Overall, when comparing the most preferred vaccine brand among the Saudi Arabian population, Pfizer (65.4%, n = 308) was shown to be more popular than the AstraZeneca vaccine (6.1%, n = 29), and 28.5% (n = 134) reported that they have no preference for the available COVID-19 vaccine brands.

The main portion of our study was based on five-point Likert scale questions that described the perception and attitudes of our participants when it comes to multiple common myths and misconceptions regarding the COVID-19 vaccine. More than half of our respondents (54.8%, n = 256) strongly agreed that COVID-19 vaccines can reduce the severity of a COVID-19 infection. Moreover, most of our respondents (61.4%, n = 289) answered “strongly disagree” when presented with the statement: “COVID-19 vaccines install microchips in the body.” The rest of the statements, as well as the corresponding response rates, are represented in Table 2.

**Table 2:** Frequency and percentages of the perception and attitudes toward COVID-19 vaccines questions (n = 471)

Perception and attitudes questions	Strongly agree, No. (%)	Agree, No. (%)	Neither agree nor disagree, No. (%)	Disagree, No. (%)	Strongly disagree, No. (%)
COVID-19 vaccines can protect you from acquiring a COVID-19 infection.	108 (22.9%)	159 (33.8%)	56 (11.9%)	86 (18.3%)	62 (13.2%)
COVID-19 vaccines can reduce the severity of a COVID-19 infection.	256 (54.8%)	158 (33.5%)	30 (6.4%)	7 (1.5%)	18 (3.8%)
COVID-19 vaccines can prevent you from transmitting COVID-19 to others.	72 (15.3%)	99 (21.0%)	126 (26.8%)	102 (21.7%)	72 (15.3%)
I can get a COVID-19 infection from the vaccine.	28 (5.9%)	44 (9.3%)	81 (17.2%)	137 (29.1%)	181 (38.4%)
COVID-19 vaccines can contribute to ending the COVID-19 pandemic.	197 (41.8%)	157 (33.3%)	76 ( 16.1%)	15 (3.2%)	26 (5.5%)
COVID-19 vaccines are not needed if you have already been infected with COVID-19.	45 (9.6%)	55 (11.7%)	121 (25.7%)	157 (33.3%)	93 (19.7%)
COVID-19 vaccines can cause infertility.	8 (1.7%)	15 (3.2%)	135 (28.7%)	136 (28.9%)	177 (37.6%)
COVID-19 vaccines install microchips in the body.	6 (1.3%)	11 (2.3%)	59 (12.5%)	106 (22.5%)	289 (61.4%)
COVID-19 vaccines can cause blood clots.	36 (7.6%)	133 (28.2%)	186 (39.5%)	72 (15.3%)	44 (9.3%)
COVID-19 vaccines can cause autism.	15 (3.2%)	16 (3.4%)	132 (28.0%)	117 (24.8%)	191 (40.6%)
COVID-19 vaccines can change your DNA.	12 (2.5%)	25 (5.3%)	115 (24.4%)	109 (23.1%)	210 (44.6%)
COVID-19 vaccines can cause seizures.	19 (4.0%)	27 (5.7%)	165 (35.0%)	115 (24.4%)	145 (30.8%)
COVID-19 vaccines can cause a heart attack.	18 (3.8%)	47 (10.0%)	185 (39.3%)	108 (22.9%)	113 (24.0%)
COVID-19 vaccines went through enough testing and development.	64 (13.6%)	116 (24.6%)	139 (29.5%)	97 (20.6%)	55 (11.7%)
COVID-19 is dangerous enough for health to risk taking a vaccine against it.	140 (29.7%)	101 (21.4%)	76 (16.1%)	77 (16.3%)	77 (16.3%)
Acquiring immunity by getting infected with COVID-19 is better than acquiring immunity by getting a vaccine.	50 (10.6%)	72 (15.3%)	139 (29.5%)	133 (28.2%)	77 (16.3%)
I would take additional doses (also known as booster shots) of the COVID-19 vaccine if required.	161 (34.2%)	147 (31.2%)	86 (18.3%)	36 (7.6%)	41 (8.7%)

When asked, “Which of the following statements describes your views on vaccinations in general?”, 83.2% agreed that vaccination is important. The chi-square analysis did not show statistical significance based on gender (P = 0.779), nationality (P = 0.211), highest degree completed (P = 0.424), and household income (P = 0.993). Statistically significant results are described in Table 3.

**Table 3:** Chi-square analysis of the question: “Which of the following statements describes your views on vaccinations in general?” * Statistically significant difference at P < 0.05.

Age group	I agree that vaccination is important. No. (%)	I am neutral. No. (%)	I am against vaccination. No. (%)	𝝌^2^	P-value
18-24	211 (88.3%)	25 (10.5%)	3 (1.3%)	(8, N = 471) = 17.329	0.027*
25-34	37 (72.5%)	13 (25.5%)	1 (2.0%)		
35-44	88 (83.0%)	16 (15.1%)	2 (1.9%)		
45-54	44 (71.0%)	15 (24.2%)	3 (4.8%)		
55+	12 (92.3%)	1 (7.7%)	0 (0.0)		
Total	392 (83.3%)	70 (14.8%)	9 (1.9%)	471 (100.0%)	
City of residence	I agree that vaccination is important. No. (%)	I am neutral. No. (%)	I am against vaccination. No. (%)	𝝌^2^	P-value
Riyadh	364 (84.8%)	58 (13.5%)	7 (1.6%)	(2, N = 471) = 9.313	0.010*
Other	28 (66.7%)	12 (28.6%)	2 (4.8%)		
Total	392 (83.3%)	70 (14.8%)	9 (1.9%)	471 (100.0%)	
Do you work or study in the healthcare field?	I agree that vaccination is important. No. (%)	I am neutral. No. (%)	I am against vaccination. No. (%)	𝝌^2^	P-value
Yes	223 (88.5%)	29 (11.5%)	0 (0.0%)	(2, N = 471) = 16.264	0.0001*
No	169 (77.2%)	41 (18.7%)	9 (4.1%)		
Total	392 (83.3%)	70 (14.8%)	9 (1.9%)	471 (100.0%)	
Do you usually take the seasonal influenza vaccine (flu shot)?	I agree that vaccination is important. No. (%)	I am neutral. No. (%)	I am against vaccination. No. (%)	𝝌^2^	P-value
Yes	80 (96.4%)	3 (3.6%)	0 (0.0%)	(4, N = 471) = 39.186	0.0001*
Sometimes	108 (95.6%)	5 (4.4%)	0 (0.0%)		
No	204 (74.2%)	62 (22.5%)	9 (3.3%)		
Total	392 (83.3%)	70 (14.8%)	9 (1.9%)	471 (100.0%)	

When asked, “Out of the following vaccines available locally, which do you prefer the most?”, a chi-square analysis showed no statistical significance based on gender (P = 0.394), age group (P = 0.070), nationality (P = 0.174), city of residence (P = 0.078), association with the field of health care (P = 0.136), the highest degree completed (P = 0.337), household income (P = 0.298), and seasonal flu vaccination (P = 0.546).

In general, the independent t-test analysis of the Likert scale question did not show statistical significance based on gender or nationality. However, those who work or study in the healthcare field showed significant P-values when presented with the following statements: COVID-19 can reduce the severity of a COVID-19 infection (P = 0.010), can contribute to ending the COVID-19 pandemic (P = 0.028), can cause blood clots (P = 0.001), can cause autism (P = 0.015), can change your DNA (P = 0.029), and can cause a heart attack (P = 0.027). Furthermore, the city of residence showed statistical significance in relation to the following statements: COVID-19 vaccines can reduce the severity of a COVID-19 infection (P = 0.009), can give me a COVID-19 infection (P = 0.036), can cause infertility (P = 0.008), can cause autism (P = 0.007), can cause seizures (P = 0.033), and can cause a heart attack (P = 0.031). Data are described in Table 4 showing mean values as well as t-test analysis results.

**Table 4:** Independent t-test analysis of perception and attitudes toward COVID-19 vaccines questions * Statistically significant difference at P < 0.05.

Do you work or study in the healthcare field? (n = 471)	Yes, mean ± SD	No, mean ± SD	T-test	P-value
COVID-19 vaccines can protect you from acquiring a COVID-19 infection.	3.412 ± 1.352	3.278 ± 1.364	1.069	0.285
COVID-19 vaccines can reduce the severity of a COVID-19 infection.	4.444 ± 0.936	4.219 ± 0.951	2.584	0.010*
COVID-19 vaccines can prevent you from transmitting COVID-19 to others.	3.031 ± 1.293	2.949 ± 1.278	0.690	0.491
I can get a COVID-19 infection from the vaccine.	2.083 ± 1.242	2.232 ± 1.151	1.348	0.176
COVID-19 vaccines can contribute to ending the COVID-19 pandemic.	4.131 ± 1.046	3.908 ± 1.145	2.200	0.028*
COVID-19 vaccines are not needed if you have already been infected with COVID-19.	2.496 ± 1.179	2.675 ± 1.226	1.620	0.107
COVID-19 vaccines can cause infertility.	1.956 ± 0.954	2.105 ± 0.987	1.659	0.098
COVID-19 vaccines install microchips in the body.	1.539 ± 0.866	1.662 ± 0.906	1.497	0.135
COVID-19 vaccines can cause blood clots.	3.250 ± 1.050	2.917 ± 1.028	3.456	0.001*
COVID-19 vaccines can cause autism.	1.928 ± 1.094	2.164 ± 0.990	2.436	0.015*
COVID-19 vaccines can change your DNA.	1.881 ± 1.068	2.095 ± 1.051	2.196	0.029*
COVID-19 vaccines can cause seizures.	2.194 ± 1.110	2.374 ± 1.047	1.801	0.072
COVID-19 vaccines can cause a heart attack.	2.365 ± 1.090	2.584 ± 1.051	2.214	0.027*
COVID-19 vaccines went through enough testing and development.	3.142 ± 1.251	3.004 ± 1.155	1.247	0.213
COVID-19 is dangerous enough for health to risk taking a vaccine against it.	3.289 ± 1.490	3.351 ± 1.420	0.459	0.646
Acquiring immunity by getting infected with COVID-19 is better than acquiring immunity by getting a vaccine.	2.817 ± 1.223	2.684 ± 1.187	1.189	0.235
I would take additional doses (also known as booster shots) of the COVID-19 vaccine if required.	3.813 ± 1.278	3.666 ± 1.205	1.277	0.202
City of residence	Riyadh Mean ± SD	Other Mean ± SD	T-test	P-value
COVID-19 vaccines can protect you from acquiring a COVID-19 infection.	3.377 ± 1.350	3.071 ± 1.420	1.396	0.163
COVID-19 vaccines can reduce the severity of a COVID-19 infection.	4.375 ± 0.902	3.976 ± 1.297	2.616	0.009*
COVID-19 vaccines can prevent you from transmitting COVID-19 to others.	2.983 ± 1.288	3.095 ± 1.265	0.536	0.592
I can get a COVID-19 infection from the vaccine.	2.116 ± 1.178	2.523 ± 1.383	2.103	0.036*
COVID-19 vaccines can contribute to ending the COVID-19 pandemic.	4.053 ± 1.056	3.761± 1.445	1.646	0.100
COVID-19 vaccines are not needed if you have already been infected with COVID-19.	2.575 ± 1.210	2.619 ± 1.146	0.222	0.824
COVID-19 vaccines can cause infertility.	1.988 ± 0.933	2.404 ± 1.250	2.668	0.008*
COVID-19 vaccines install microchips in the body.	1.587 ± 0.883	1.690 ± 0.923	0.719	0.473
COVID-19 vaccines can cause blood clots.	3.074 ± 1.047	3.309 ± 1.092	1.382	0.168
COVID-19 vaccines can cause autism.	1.997 ± 1.026	2.452 ± 1.233	2.688	0.007*
COVID-19 vaccines can change your DNA.	1.960 ± 1.037	2.190 ± 1.311	1.338	0.182
COVID-19 vaccines can cause seizures.	2.244 ± 1.067	2.619 ± 1.208	2.143	0.033*
COVID-19 vaccines can cause a heart attack.	2.433 ± 1.056	2.809 ± 1.234	2.167	0.031*
COVID-19 vaccines went through enough testing and development.	3.107 ± 1.202	2.785 ± 1.240	1.649	0.100
COVID-19 is dangerous enough for health to risk taking a vaccine against it.	3.331 ± 1.442	3.190 ± 1.611	0.596	0.551
Acquiring immunity by getting infected with COVID-19 is better than acquiring immunity by getting a vaccine.	2.736 ± 1.212	2.952 ± 1.146	1.106	0.269
I would take additional doses (also known as booster shots) of the COVID-19 vaccine if required.	3.769 ± 1.207	3.500 ± 1.581	1.338	0.182

The one-way ANOVA of the perception and attitudes toward COVID-19 vaccines questions portrayed significance in relation to the following factors: age group, highest degree completed, household income, and seasonal flu vaccination. The Tukey post hoc test for multiple comparisons between groups was conducted on these groups and portrayed in a compact letter display, as shown in Table 5.

**Table 5:** One-way ANOVA of perception and attitudes toward COVID-19 vaccines questions * Statistically significant difference at P < 0.05. Means that do not share superscripts differ by P < 0.05 according to Tukey's HSD.

Age group	18-24, mean ± SD	25-34, mean ± SD	35-44, mean ± SD	45-54, mean ± SD	55+, mean ± SD		F-value	P-value
I can get a COVID-19 infection from the vaccine.*	2.209 ± 1.246^a^	2.509 ± 1.376^a^	2.028 ± 1.125^a^	1.951 ± 1.015^a^	1.692 ± 0.630^a^		2.483	0.043*
COVID-19 vaccines can cause autism.	1.861 ± 1.097^a^	2.294 ± 1.221^ab^	2.229 ± 0.897^b^	2.193 ± 0.920^ab^	2.000 ± 0.816^ab^		3.698	0.006*
COVID-19 is dangerous enough for health to risk taking a vaccine against it.	3.146 ± 1.483^a^	3.509 ± 1.391^ab^	3.632 ± 1.389^b^	3.145 ± 1.435^ab^	4.000 ± 1.354^ab^		3.271	0.012*
Highest degree completed		Have not completed a high school diploma, mean ± SD	High school diploma, mean ± SD	Bachelor’s degree, mean ± SD	Postgraduate degree, mean ± SD		F-value	P-value
COVID-19 vaccines are not needed if you have already been infected with COVID-19.		4.000 ± 1.154^a^	2.582 ± 1.185^abc^	2.684 ± 1.240^ab^	2.288 ± 1.093^c^		4.169	0.006*
COVID-19 vaccines install microchips in the body.		2.500 ± 1.914^a^	1.550 ± 0.874^a^	1.689 ± 0.933^a^	1.455 ± 0.705^a^		3.045	0.029*
Household income		<3000 Riyals, mean ± SD	3000-9999 Riyals, mean ± SD	10000-24999 Riyals, mean ± SD	>25000 Riyals, mean ± SD		F-value	P-value
I can get a COVID-19 infection from the vaccine.		2.410 ± 1.271^a^	2.218 ± 1.272^a^	2.305 ± 1.158^a^	2.004 ± 1.186^a^		2.633	0.049*
COVID-19 vaccines are not needed if you have already been infected with COVID-19.		2.871 ± 1.360^ab^	2.618 ± 1.178^ab^	2.829 ± 1.182^a^	2.372 ± 1.161^b^		5.277	0.001*
COVID-19 vaccines install microchips in the body.		2.000 ± 1.169^a^	1.800 ± 1.095^ab^	1.723 ± 0.846^ac^	1.406 ± 0.752^d^		8.626	0.0001*
Do you usually take the seasonal influenza vaccine (flu shot)?		Yes Mean ± SD	Sometimes Mean ± SD	No Mean ± SD	F-value	P-value		
COVID-19 vaccines can protect you from acquiring a COVID-19 infection.		3.710 ± 1.401^a^	3.469 ± 1.165^ab^	3.192 ± 1.397^b^	5.301	0.005*		
I can get a COVID-19 infection from the vaccine.		1.843 ± 1.029^ab^	1.964 ± 1.109^b^	2.323 ± 1.258^c^	7.093	0.001*		
COVID-19 vaccines can contribute to ending the COVID-19 pandemic.		4.180 ± 0.977^ab^	4.327 ± 0.900^b^	3.858 ± 1.173^c^	8.560	0.0001*		
COVID-19 vaccines are not needed if you have already been infected with COVID-19.		2.445 ± 1.139^ab^	2.380 ± 1.112^a^	2.701 ± 1.246^b^	3.514	0.031*		
COVID-19 vaccines can cause infertility.		1.807 ± 0.861^ab^	1.858 ± 0.874^b^	2.160 ± 1.019^c^	6.551	0.002*		
COVID-19 vaccines can cause autism.		1.903 ± 0.903^ab^	1.778 ± 0.923^b^	2.185 ± 1.113^a^	6.967	0.001*		
COVID-19 vaccines can change your DNA.		1.795 ± 0.984^ab^	1.725 ± 0.918^b^	2.141 ± 1.116^c^	7.874	0.0001*		
COVID-19 vaccines can cause seizures.		2.132 ± 0.997^ab^	2.070 ± 1.041^a^	2.407 ± 1.111^b^	4.843	0.008*		
Acquiring immunity by getting infected with COVID-19 is better than acquiring immunity by getting a vaccine.		2.506 ± 1.182^ab^	2.477 ± 1.118^b^	2.945 ± 1.217^c^	8.423	0.0001*		
I would take additional doses (also known as booster shots) of the COVID-19 vaccine if required.		4.096 ± 1.185^a^	3.831 ± 1.101^ab^	3.603 ± 1.298^b^	5.448	0.005*		

## Discussion

The main objective of this study is to investigate the common myths, misconceptions, and attitudes toward the COVID-19 vaccine in the Saudi Arabian community and the frequency with which they are present. This evaluation will help us interpret the impact of these misconceptions on the willingness of the population to get vaccinated, in hopes of eradicating this novel virus. Spreading awareness of the importance of getting vaccinated will help control the spread of COVID-19, as well as reduce the severity of the disease and mortality. Generally, the findings of our study demonstrate a good level of awareness of vaccines, and general acceptance of the specific COVID-19 vaccine has been observed. This was evident in our results, as most of our respondents (83.2%) agreed that vaccines are important. Similarly, a cross-sectional study in China (n = 3541) has shown that 83.3% of the Chinese population has an intention to receive the COVID-19 vaccine [18]. Similarly, a European study (n = 7644) that included participants from Denmark, Germany, France, Italy, Portugal, the Netherlands, and the UK has shown a 73.9% acceptance of the COVID-19 vaccination [19].

Our study showed that the Saudi Arabian population has widely accepted COVID-19 vaccines, as 91.5% of our respondents mentioned that they had already taken the first dose; however, there was still bias when it comes to the preference for the vaccine brand, as we still found that a large portion of the population prefers Pfizer (65.4%) over other vaccines. This may be attributed to the rare side effects reported (thrombosis and thrombocytopenia) of the Oxford-AstraZeneca vaccine, which can occur in about four to six people per million after vaccination [20]. Similar to our study, a previously published cross-sectional study in Jordan (n = 1887) revealed that acceptance of the Pfizer/BioNTech vaccine and knowledge of it were significantly better than all other vaccines [21]. Another cross-sectional study conducted in the US (n = 1027) reflected major misconceptions regarding certain vaccines; for example, most Americans underestimated the size of the Pfizer/BioNTech and Moderna clinical trials. Only 15.0% of the respondents (95% CI: 12.2% to 17.7%) correctly responded that the two combined trials had enrolled more than 50,000 participants.

Despite the wide acceptance of the vaccine reflected in our results, only 54.8% of our respondents strongly agreed that COVID-19 vaccines can reduce the severity of a COVID-19 infection, and 41.8% agreed that COVID-19 vaccines can contribute to ending the pandemic. These percentages are not ideal and should be higher considering the immense efforts done by the Saudi government to raise awareness regarding COVID-19 vaccines. Some of our data showed the need for more vaccine education specifically aimed at groups with lower education levels. This is apparent in the significance of agreement with statements such as “COVID-19 vaccines are not needed if you have already been infected with COVID-19” and “COVID-19 vaccines install microchips in the body” among groups with lower degrees of education. These results align with a study conducted in Japan (n = 2956) where lower education levels were significantly associated with a reluctance to vaccinate against COVID-19 [22].

If we were to look at other published studies, a noticeable number of articles were published depicting a lack of awareness of the importance of vaccines. A cross-sectional study in Jordan (n = 3100) identified Jordan as one of the lowest countries in the acceptance of COVID-19 vaccines. A considerable percentage of the population of Jordan (36.3%) indicated a refusal to get vaccinated, while 26.3% were unsure and confused [23]. Similarly, another rapid qualitative assessment in South Africa revealed that the South African population was aware of the transmission of COVID-19; however, the majority refused the vaccine as misconceptions about its efficacy were more prominent in the data [24].

When we further analyzed the data based on the baseline characteristics of our respondents, our study revealed a significantly higher prevalence of misconceptions among people living outside of Riyadh versus people living inside of Riyadh. That is evident in the increased agreement to statements such as “I can get COVID-19 infection from the vaccine,” “COVID-19 vaccine causes infertility,” and “COVID-19 vaccines can cause autism.” Furthermore, when asked if vaccination is important, 84.8 % (n = 364) of participants living in Riyadh agreed with the statement, compared to only 66.7% (n = 28) of participants living outside of Riyadh. Similarly, a cross-sectional study conducted in Shanghai, China (n = 1288) revealed that individuals living in the outer suburbs (β: 0.13; 95% CI: 0.01, 0.25) and rural non-locals (β: 0.10; 95% CI: 0.02, 0.18) had a greater lack of confidence in vaccines compared to their urban or local counterparts, respectively [25].

Sociodemographic factors of age, city of residence, household income, and level of education were significant in determining the willingness to accept COVID-19 vaccines, such as studies carried out in the UK (n = 1500), France (n = 3259), the US (n = 672), China (n = 2058), and Japan (n = 2956) [22,26-29].

Our study revealed significantly greater acceptance and willingness to be vaccinated among healthcare workers (88.5%) compared to non-healthcare workers (77.2%). Similarly, a cross-sectional study conducted in China (n = 2580) revealed that 76.98% of healthcare workers accepted the COVID-19 vaccine, while only 56.19% of non-healthcare workers accepted the COVID-19 vaccine [30].

If we looked at other factors, our study did not show statistical significance between gender and COVID-19 vaccination. On the other hand, a study by Malik et al. in the United States (n = 672) showed that men (72%) compared to women were more likely to accept the vaccine [27]. Moreover, in our study, there was a significance between those who took the seasonal influenza vaccine and their general perception and attitudes toward the COVID-19 vaccine. Similarly, Sherman et al. revealed in a study conducted in the UK (n = 1500) that an increased probability of being vaccinated for COVID-19 was significantly associated with having been vaccinated for influenza last winter [29].

To provide the correct information regarding vaccines to the Saudi population, it is of utmost importance to know from which source it is most used and trusted. Our study revealed that the most trusted source of information on the COVID-19 vaccine for most of our participants (86.4%) was the Saudi Ministry of Health. A multimethod study conducted in the UK (n = 1252) revealed that survey participants would receive the vaccine for themselves or their children if it was recommended by the government [31]. A global survey (n = 13,466) of possible acceptance of COVID-19 revealed that respondents who reported higher levels of trust in government information were more likely to accept a vaccine and take their employer's advice to do so, which we believe could be a contributing factor to the general acceptability of vaccines reported in our study [32].

Study limitations

This study was conducted by circulating an online questionnaire to collect data from a convenient sample, thereby limiting people without internet access and affecting the generalizability of the study. In addition to that, only 38.4% of the respondents were of Saudi nationality, compared to 68.9% of the general population of Saudi Arabia [16]. Furthermore, 91.1% of the respondents reside in Riyadh; meanwhile, only 25.5% of the general population resides in the Riyadh province [16].

## Conclusions

In conclusion, since COVID-19 vaccines are being newly introduced, public health officials should increase awareness measures regarding COVID-19 vaccines to limit myths and misconceptions. Such awareness campaigns can be carried out through social media, as well as educational booths and advertisements. Since our study has evidently shown a preference for the Pfizer vaccine, another suggestion may be to increase the availability of this vaccine in hopes of increasing vaccination rates in the kingdom. Future studies are needed among the Saudi population to better understand their perception and attitudes toward COVID-19 vaccines to help further prevent and, we hope, eradicate this pandemic.

## Appendices

The survey used in our study

Questionnaire: 15 Questions

Gender:

Female

Male

Age:

What is your nationality?

Saudi

Non-Saudi

City of residence:

Riyadh

Jeddah

Dammam

Medina

Mecca

Tabuk

Taif

Other: ________

Do you work or study in the healthcare field (medicine, pharmacy, nursing, healthcare management, etc.)?

Yes

No

What is the highest degree or level of school you have completed? (If you are currently enrolled in school, please indicate the highest degree you have received)

Have not completed a high school diploma

High school diploma

Bachelor's degree

Master's degree or higher

Other:_________

What is your monthly household income?

<3,000 SAR

3,000-9,999 SAR

10,000-25,000 SAR

>25,000 SAR

Are you up to date with all your childhood vaccinations?

Yes

No

I do not know

Do you take the yearly seasonal influenza vaccine (flu shot)?

Yes

No

Sometimes

Which of the following statements describes your views on vaccination in general?

I agree that vaccination is important

I am neutral

I am against vaccination

Which of the following is your most trusted source of information regarding the COVID-19 vaccine? (You can select more than one option)

Social media

The Saudi MOH

Awareness campaigns

Word of mouth

Television

Your trusted doctor

Other: __________

In regards to the COVID-19 vaccine, which of the following statements apply to you at this time?

I have already received at least one dose of the vaccine

I have an appointment to receive the vaccine

I am still waiting for the availability of an appointment to reserve it

I am still waiting for more time to be sure of the safety and effectiveness of the vaccine

I am not eligible to take the vaccine

I do not intend to take the vaccine

Out of the following vaccines available locally, which one do you prefer the most? 

AstraZeneca vaccine

Pfizer vaccine

I have no preference

Why do you prefer the vaccine chosen in the question above? (Optional)

**Table 6:** Please choose if you agree/disagree with the following statements

	Strongly disagree	Disagree	Neutral	Agree	Strongly agree
COVID-19 vaccines can protect you from acquiring a COVID-19 infection.					
COVID-19 vaccines can reduce the severity of a COVID-19 infection.					
COVID-19 vaccines can prevent you from transmitting COVID-19 to others.					
You can get COVID-19 infection from the vaccine.					
COVID-19 vaccines can contribute to ending the COVID-19 pandemic.					
COVID-19 vaccines are not needed if you have already been infected with COVID-19.					
COVID-19 vaccines can cause infertility.					
COVID-19 vaccines install microchips in the body.					
COVID-19 vaccines can cause blood clots.					
COVID-19 vaccines can cause autism.					
COVID-19 vaccines can change your DNA.					
COVID-19 vaccines can cause seizures.					
COVID-19 vaccines can cause a heart attack.					
COVID-19 vaccines are safe enough.					
COVID-19 vaccines went through enough testing and development.					
COVID-19 is dangerous enough to health to risk taking a vaccine against it.					
Acquiring immunity by getting infected with COVID-19 is better than acquiring immunity by getting a vaccine.					

Do you have any comments or suggestions? (Optional)
